# Breaking Barriers: Immune Checkpoint Inhibitors in Breast Cancer

**DOI:** 10.3390/pharmaceutics18010034

**Published:** 2025-12-26

**Authors:** Bartosz Dmuchowski, Witold Wit Hryniewicz, Igor Barczak, Kacper Fręśko, Zuzanna Szarzyńska, Hubert Węclewski, Jan Kazimierz Ślężak, Paula Dobosz, Hanna Gryczka

**Affiliations:** 1 Faculty of Medicine, Poznan University of Medical Sciences, 61-701 Poznan, Polandhryniewiczwitek@wp.pl (W.W.H.); hubert.weclewski2003@gmail.com (H.W.); jankazimierzslezak@gmail.com (J.K.Ś.); 2 Department of Patomorphology, Poznan University of Medical Sciences, 61-701 Poznan, Poland

**Keywords:** breast cancer, immunotherapy, checkpoint inhibitors, PD-1/PD-L1, resistance to immunotherapy, immunotherapy biomarkers

## Abstract

Breast cancer remains the most commonly diagnosed malignancy among women worldwide and continues to pose significant therapeutic challenges, particularly in advanced and refractory disease. Although traditionally considered less immunogenic compared with other solid tumours, growing evidence demonstrates that subsets of breast cancer, particularly triple-negative and HER2-positive subtypes, exhibit immune-responsive features. This recognition has spurred the development and clinical evaluation of immunotherapeutic strategies, with immune checkpoint inhibitors (ICIs) emerging as the most prominent approach. This new class of drugs targeting the programmed death-1 (PD-1)/programmed death-ligand 1 (PD-L1) axis has demonstrated meaningful clinical activity in select patient populations, leading to regulatory approvals in combination with chemotherapy for advanced triple-negative breast cancer. Despite these advances, response rates remain modest, and the benefits are largely restricted to patients with PD-L1-positive tumours. Ongoing studies are evaluating predictive biomarkers, optimal treatment combinations, and mechanisms of resistance to expand the efficacy of ICIs across broader breast cancer subtypes. Furthermore, novel checkpoint targets such as cytotoxic T-lymphocyte-associated protein 4 (CTLA-4), lymphocyte-activation gene 3 (LAG-3), and T cell immunoreceptor with immunoglobulin and immunoreceptor tyrosine-based inhibitory motif domains (TIGIT) are under investigation, with the potential to enhance or complement PD-1/PD-L1 blockade. This review summarises the current state of knowledge on breast cancer immunotherapy with an emphasis on ICIs, highlighting key clinical trial findings, as well as emerging biomarkers of response, and strategies to overcome therapeutic resistance, if cancer cells eventually develop resistance. By integrating preclinical insights with clinical progress, we aim to provide a comprehensive overview of the evolving role of checkpoint blockade in breast cancer and outline future directions to optimise patient outcomes.

## 1. Introduction

Breast cancer is the most common malignant tumour in women, making it a major concern for doctors, patients, their families, and society as a whole [1]. It predominantly affects women, in whom it represents one of the most common malignant tumours, with incidence increasing with age. More than 80% of breast cancer cases are diagnosed in patients over the age of 50 [2,3]. In addition to age, risk factors for breast cancer include genetic predisposition, female gender, a family history of breast cancer, previous radiotherapy, exposure to diethylstilbestrol, use of hormonal contraceptives, hormone replacement therapy, excessive alcohol consumption, overweight and obesity, nulliparity, early menarche, late menopause, and lack of physical activity [3]. It is worth noting that although the risk of developing breast cancer increases with age, younger patients tend to receive a diagnosis at more advanced stages of the disease [4].

### 1.1. Histological Types of Breast Cancer

**Figure 1:**
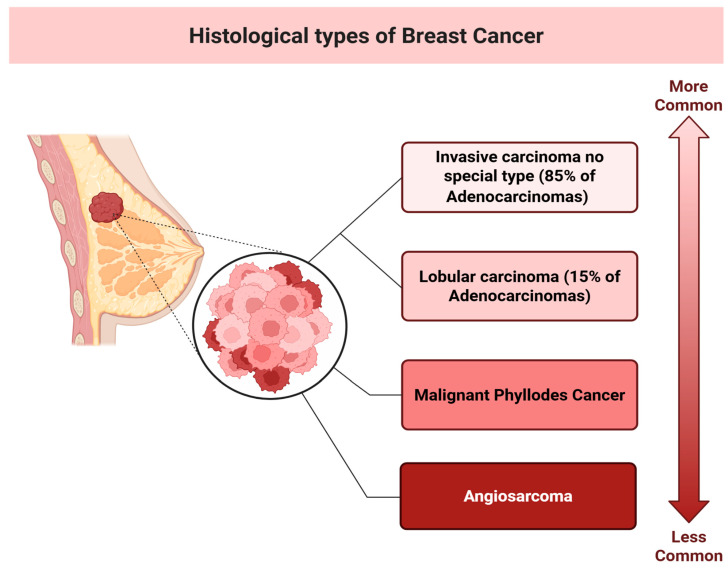
Histological types of breast cancer—this almost historical approach has been recently challenged by the advanced genomic analyses and multi-omics investigations. Created with BioRender.com.

Most cases of breast cancer are adenocarcinomas, with 85% of adenocarcinoma cases being ductal adenocarcinomas and 15% being lobular adenocarcinomas. Other types of breast cancer include Paget’s disease of the breast and inflammatory breast cancer. Rarely, sarcomas such as angiosarcoma and malignant phyllodes cancer may also occur in the breast [2]. Triple-negative breast cancer (TNBC) is a unique form of breast cancer, characterised by the absence of progesterone and oestrogen receptors, as well as human epidermal growth factor receptor 2 (HER2) [5]. TNBC accounts for 10–15% of all breast cancer cases. It is associated with a high risk for metastasis, poor prognosis, and a high recurrence rate. Its diagnosis is based on imaging and immunohistochemistry, which enables the implementation of personalised therapy for patients with TNBC [6]. The assignment to a certain stage depends on the tumour’s size, histological type, and affected area. Stage 0 indicates a preinvasive cancer, meaning it is confined to its site of origin with no evidence of invasion into surrounding tissues. Stages 1, 2, 3, and 4 indicate invasive cancer, with stage 4 being characterised by the presence of distant metastases to other organs, such as the lungs [5]. Traditionally, breast cancer classification relied on histological types, as depicted in Figure 1; however, this approach is now considered somewhat outdated. Advances in genomic and transcriptomic profiling have revealed that breast cancer is a highly heterogeneous group of malignancies. Molecular subtyping has identified dozens of genetic and transcriptomic subgroups, each with distinct biological behaviour and therapeutic vulnerabilities. Even TNBC, once considered a uniform entity, is now subdivided into multiple molecular subtypes, which differ in prognosis and response to targeted therapies [7]. This molecular understanding increasingly guides treatment strategies, enabling precision medicine and individualised therapy approaches [8].

### 1.2. Prognosis

The key prognostic factors in breast cancer include the stage of the disease at diagnosis, tumour grade, and lymph node status [9]. The prognosis of breast cancer largely depends on the tumour microenvironment (TME) classification; however, this system does not account for all relevant variables, such as the molecular characteristics of the tumour [10]. The course and outcome of the disease are influenced by multiple factors. One such factor is the presence of specific genetic mutations. Carriers of *BRCA1* and *BRCA2* mutations have been shown to exhibit poorer prognosis compared to non-carriers [11]. According to NCCN guidelines, individuals carrying *BRCA1* mutations face a 60–72% lifetime risk of developing breast cancer—most often TNBC—compared with approximately 12% in the general population [12]. Another important prognostic factor is the age at which the cancer is diagnosed. Breast cancer diagnosed in women under 40 years of age is associated with a worse prognosis than in older patients [4]. Patients’ race also influences disease progression. Studies have shown that breast cancer tends to have a poorer prognosis in black women compared to white women, which is attributed to a combination of tumour biology, socioeconomic conditions, reproductive patterns, access to healthcare, and other factors. Furthermore, TNBC in black women tends to follow a more aggressive course than the same subtype in white women [13]. It is also worth noting that, despite histological similarities, breast cancer in men generally has a worse prognosis than in women. This is because male breast cancer is typically diagnosed at a later stage, and the average age of onset is higher in men, which is associated with an increased likelihood of complications such as cardiovascular diseases [14]. Recently, tumour-infiltrating lymphocytes (TILs) have gained increasing attention as a prognostic indicator. A recent meta-analysis confirmed that patients with higher TIL levels exhibit significantly improved disease-free survival, overall survival, and pathological complete response [15]. Moreover, patients with higher TIL levels show a better response to neoadjuvant breast cancer therapy [16].

### 1.3. Breast Cancer Treatment Methods

Breast cancer treatment includes many options—immunotherapy, surgery, chemotherapy, radiotherapy, hormone therapy, and vaccines. Early-stage breast cancer is commonly treated by systemic neoadjuvant therapy. However, the appropriate use of certain methods and agents depends on the subtype and stage of a specific cancer. Choosing between different agents and methods is essential to achieve both therapeutic success and ensure good quality of life [17,18]. This paper will focus on immunotherapy, especially immune checkpoint inhibitors (ICIs), which are a novel therapeutic method in breast cancer treatment.

#### 1.3.1. Surgical Treatment

Surgery remains the main therapeutic method in early cancer stages. Its role is crucial in conjunction with chemotherapy, radiotherapy, and immunotherapy [19]. It is performed in order to remove the cancerous tissue—breast cancer cells, which modulate the tumour microenvironment [20], and cancer-associated adipocytes, which can promote the invasion and metastasis of breast cancer [21].

#### 1.3.2. Radiotherapy

Radiotherapy induces direct deoxyribonucleic acid (DNA) damage to tumour cells. Treatment of local tumours may induce a systemic antitumour effect known as the abscopal effect [22]. A combination of immunotherapy and radiotherapy may enhance systemic immunogenic response [23]. Some effects caused by radiation can also be detrimental, such as recruitment of regulatory T cells [24], immunosuppressive cytokines, and chemokines [25], and up-regulation of PD-L1 [26]. The use of ICIs, such as CTLA-4 inhibitors and especially PD-1 inhibitors, may be crucial to the improvement of this treatment method [27].

#### 1.3.3. Chemotherapy

Chemotherapy, as a first-line treatment, is used in metastatic TNBC. In early-stage disease, the standard of care consists of regimens based on anthracyclines and taxanes, administered sequentially or in combination, which are associated with the highest rates of complete pathological response. In metastatic TNBC, cytotoxic chemotherapy, mainly taxanes, constitutes the backbone of treatment, and the choice of regimen depends on prior therapies, the patient’s condition, and the molecular characteristics of the tumour. In recent years, combinations of ICIs with chemotherapeutic agents have been increasingly applied [28]. This combination is particularly relevant in unresectable or metastatic TNBC. Chemotherapeutics induce Immunogenic Cell Death (ICD), increase CRT exposure, and promote the release of ATP and HMGB1 [29]. The release of these damage-associated molecular patterns (DAMPs) activates the immune response and enhances the effectiveness of immunotherapy [30].

#### 1.3.4. Hormone Therapy

Hormone therapy, such as tamoxifen, aromatase inhibitors, and gonadotropin-releasing hormone (GnRH) analogues, is the mainstay of treatment for hormone-dependent breast cancer (HR+), particularly for premenopausal women [31]. Although not yet confirmed, combining hormone therapy with PD-1 inhibitors is suggested to be effective in ER+ metastatic breast cancer. Aromatase inhibitors combined with GnRH analogues modify the tumour microenvironment, increasing immune cell infiltration and activity, thereby enhancing the effectiveness of pembrolizumab [32]. Other studies suggest a possible, though unconfirmed, increase in PD-L1 expression after neoadjuvant endocrine therapy (NET). Therefore, routine use of atezolizumab following hormone therapy may be feasible in the future [33].

#### 1.3.5. Immunotherapy

The landscape of immunological treatment methods is broader than immune checkpoint inhibitors, our main topic of interest in this article. One of these innovative treatment approaches includes the use of natural or artificially engineered oncolytic viruses. Used primarily in solid cancers, they are capable of directly lysing cancer cells without harming normal tissues. Furthermore, they can induce antitumour immunity by modifying the tumour microenvironment [34,35]. Lysis of cancer cells is caused by selective infection and subsequent replication of the virus within the tumour. It should be emphasised that after lysis, the lytic cycle of the virus repeats until the virus is weakened by the host’s immune response [36]. The death of cancer cells stimulates the release of tumour-associated antigens (TAA), damage-associated molecular patterns (DAMP), and pathogen-associated molecular patterns (PAMP). These serve as signals for antigen-presenting cells to initiate an immune response [37]. Despite the promising mechanism of action, oncolytic viruses are unlikely to be used as monotherapy for breast cancer due to the lack of complete tumour regression [35].

#### 1.3.6. Vaccines

Another promising perspective in breast cancer immunotherapy has emerged in recent years, with a focus on neoantigen-targeted vaccines. They utilise various antigen delivery platforms. DNA vaccines introduce a plasmid encoding tumour-specific mutations. This leads to protein synthesis, which is subsequently presented on MHC class I and II. The downstream effect is the stimulation of T lymphocytes to attack tumour cells [38]. Dendritic cells derived from induced pluripotent stem cells (iPSC) constitute a modern system capable of simultaneous presentation of multiple epitopes, supporting the T cell response. Preclinical studies indicate that iPSC-based vaccines enriched with neoantigens effectively activate tumour-specific T lymphocytes and demonstrate antitumour activity in breast cancer models [39]. It seems that the aforementioned approaches have advantages over traditional dendritic cell vaccines. Despite clinical success, DC vaccines are limited by the need to obtain autologous patient cells, which significantly restricts large-scale production [40,41]. In recent years, the development of breast cancer immunotherapy has also included silico-designed mRNA vaccines that combine multiple neoantigens in a single construct. Preclinical studies have evaluated mRNA vaccines targeting the neoantigen CA-125 for the treatment of breast cancer, among other applications. These studies demonstrated the induction of cytotoxic T lymphocytes and IFN-gamma production, creating prospects for personalised breast cancer therapy [42].

Despite their incredible potential and interesting mechanisms of action, this manuscript cannot describe all in detail; therefore, a short summary has been presented in Table 1.

## 2. Tumour Microenvironment in Breast Cancer Immunotherapy

The tumour microenvironment encompasses cancer cells, immune cells, and both local and systemic factors and components of the immune system. The tumour microenvironment and the interactions among its components are being increasingly studied, as it is recognised as a key target of modern anticancer therapies. In cancer, the TME can be divided into cellular components, which contain cancer cells as well as various immune cells, and soluble factors, which include cytokines, chemokines, growth factors, pro-inflammatory factors, lactate, nutrients, and other substances. Additionally, the TME encompasses physical factors such as tissue architecture, hypoxia, tissue stiffness, and mechanical forces. Cellular elements are classified into three compartments: local (the tumour and its environment), regional (breast), and metastatic [48,49,50,51]. Since the main focus of our study is immunotherapy, in this discussion we will concentrate on the role of immune cells within the TME.

### 2.1. The Role of Immune Cells

**Figure 2:**
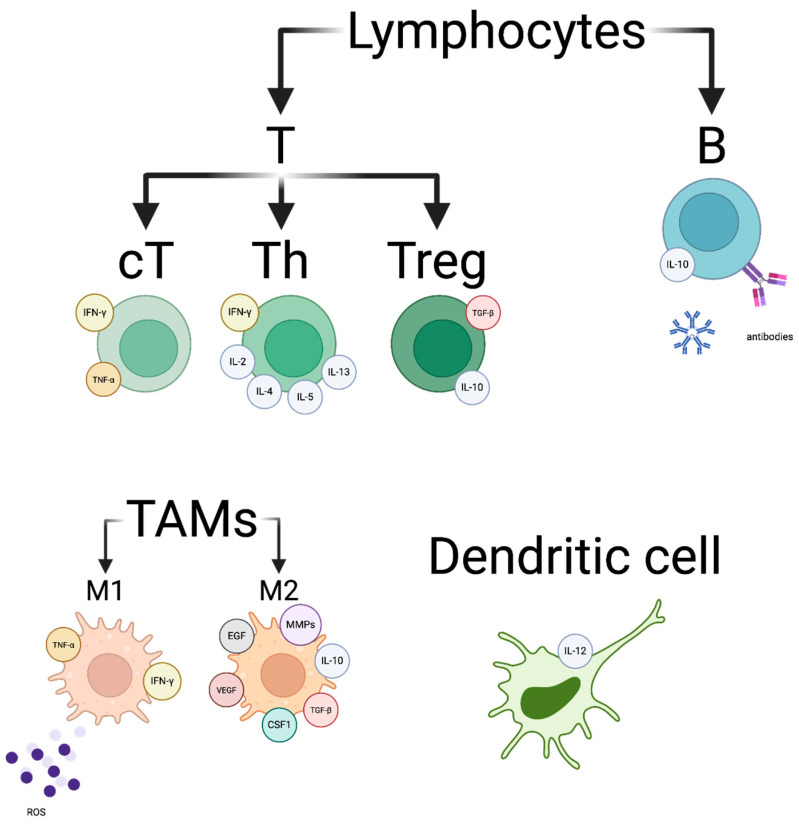
Tumour microenvironment cells and their molecules regulating antitumour immunity.

**Figure 3:**
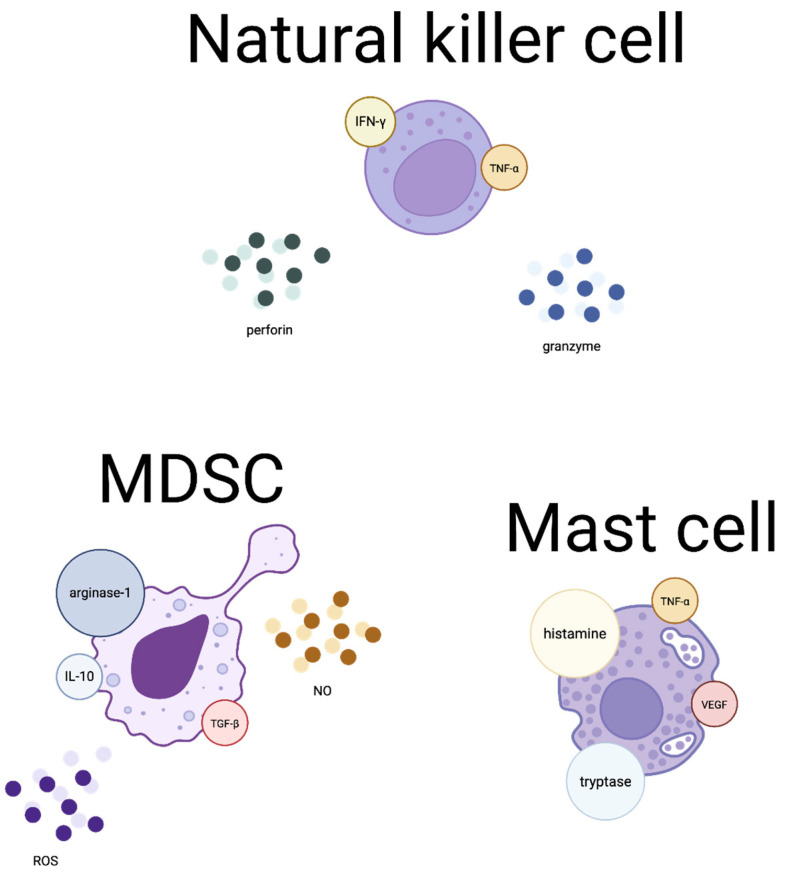
Tumour microenvironment cells and their molecules regulating antitumour immunity.

Despite relatively low immunogenicity compared to other malignant tumours, breast cancers show activity of various immune cells. A graphical representation, along with the substances they produce and their classification into immunosuppressive and immunostimulatory, is shown in Figure 2 and Figure 3.

#### 2.1.1. Tumour-Infiltrating Lymphocytes

Tumour-infiltrating lymphocytes (TILs) are lymphocytes that have migrated from the circulation into the tumour microenvironment, and their existence plays a key role in the immune response against cancer. The dominant cell populations are CD8+, CD4+, FOXP3+, and CD19+ cells [52]. In the context of ICI therapy, tumour cells may manifest increased expression of programmed death-ligand (PD-L1), resulting in reduced cytotoxic activity of TILs against the tumour [53]. The mechanism of immunotherapy consists of blocking the interaction between PD-L1 and PD-1 on the surface of cytotoxic TILs, thereby allowing them to fight the tumour. As a result, TIL density may have potential predictive value in the case of this type of therapy [54]. Depending on the molecular subtype of the tumour, varying degrees of tumour infiltration by TILs have been observed, with the highest levels seen in TNBC and HER2+ breast cancer [55]. In luminal-type cancers, the intensity of infiltration is lower than in TNBC or HER2+ breast cancer, and a higher ratio of TILs correlates with a worse prognosis [56,57]. It is hypothesised that this is related to a higher Ki67 index and a different cellular composition, with a higher proportion of FOXP3+ immunosuppressive cells and a lower proportion of cytotoxic CD8^+^ cells [58,59]. Metastases, particularly in TNBC, show lower TIL density, and their importance has not yet been fully explained [60].

#### 2.1.2. Regulatory T Cells

Regulatory T cells (Tregs) are lymphocytes with an immunosuppressive profile that physiologically preserve immune self-tolerance in the body [61]. However, their presence in the TME may influence tumour progression as a result of their immunosuppressive activity and the enablement of tumour escape from immune system control [62]. The main protein mediating the immunosuppressive activity of this cell is CTLA-4, which inhibits T cell activation; therefore, CTLA-4 inhibitors can reactivate T cells and manifest antitumour effects [63,64]. Analyses have also shown that such therapy may be even more effective in tumours with a low level of glycolysis [65]. The role of the PD-1/PD-L1 pathway in Tregs has not yet been fully defined [66]. Despite extensive research on the significance of Treg levels in breast cancer, no definitive conclusion has been reached due to conflicting results. It can be tentatively concluded that their role depends on the breast cancer subtype [67].

#### 2.1.3. Tumour-Associated Macrophages

Tumour-associated macrophages (TAMs) are macrophages residing in the TME. They constitute the most numerous population of immunosuppressive and pro-tumourigenic cells in the TME, inducing these effects through numerous mediators, direct inhibition of cytotoxic lymphocyte proliferation, recruitment of Tregs, and by promoting increased PD-L1 expression on tumour cells [68,69,70]. From the perspective of immunotherapy, the latter mechanism is of particular importance.

Anti–PD-1/PD-L1 therapy modulates, among other things, macrophage maturation, enhances polarisation toward a pro-inflammatory phenotype while reducing immunosuppressive polarisation, increases phagocytic capacity, and promotes T lymphocyte activation and proliferation [71,72].

#### 2.1.4. Myeloid-Derived Suppressor Cells

Myeloid-derived suppressor cells (MDSCs), together with TAMs, belong to the most numerous populations of immune cells within the TME. They are heterogeneous myeloid cells with immunosuppressive properties [73]. From the perspective of immunotherapy, a strong correlation has been observed between MDSC levels and the response to anti–PD-L1 or anti–CTLA-4 therapy, where elevated MDSC levels being associated with resistance to ICIs [74,75]. The combination of anti–PD-L1 and anti–CTLA-4 antibodies with MDSC depletion proved highly effective against the aggressive 4T1 TNBC model, resulting in tumour and metastasis regression with a survival rate >80% following tumour implantation [76,77].

### 2.2. The Role and Expression of Programmed Cell Death Protein 1 in Tumour and Immune Cells

Programmed death-ligand 1 (PD-L1) is a ligand of a type 1 transmembrane protein, expressed on immune cells, mainly T lymphocytes, B lymphocytes, and antigen-presenting cells (APCs) [78]. Their function is to inhibit the immune response of these cells upon binding to PD-1 receptors, which, under physiological conditions, serves a protective role against excessive reactions to foreign antigens or autoimmunity against self-antigens. Malignant tumours can modulate PD-L1 expression on the surface of TILs, leading to suppression of cytotoxic lymphocytes (Tc) responses and an increased survival of tumour cells [79]. Therefore, there are therapies aimed at blocking this axis, and research has demonstrated their effectiveness in inducing recognition and elimination of tumour cells by Tc [80]. In clinical practice, for patients who are candidates for PD-L1 inhibitor therapy, the expression levels of these receptors in tumour tissues are assessed using immunohistochemical methods [79]. In breast cancer, varying levels of PD-L1 expression are observed depending on the type. In luminal breast cancer (hormone receptor—HR+/HER2−), PD-L1 expression is seen in approximately 9% of cases in type A and 42% of cases in type B. In metastatic tumours, these figures are approximately 0–1% and 10–12%, respectively [81]. In HER2-positive breast cancer, immunoreactivity is observed in approximately 30% of cases in the primary tumour and around 9–10% of cases in metastatic tumours [82,83]. The most interesting aspect regarding TNBC, which encompasses multiple molecular subtypes, is that only the immunomodulatory subtype exhibits PD-L1 expression. PD-L1 expression in this TNBC subtype is present in approximately 45–55% of primary tumours and in around 35% of metastatic cases, and it differs depending on the metastatic location —being significantly lower in liver, skin, and bone metastases, while in other locations the decrease is less pronounced [84,85,86].

### 2.3. Immunosuppressive vs. Immunostimulatory Factors

**Figure 4:**
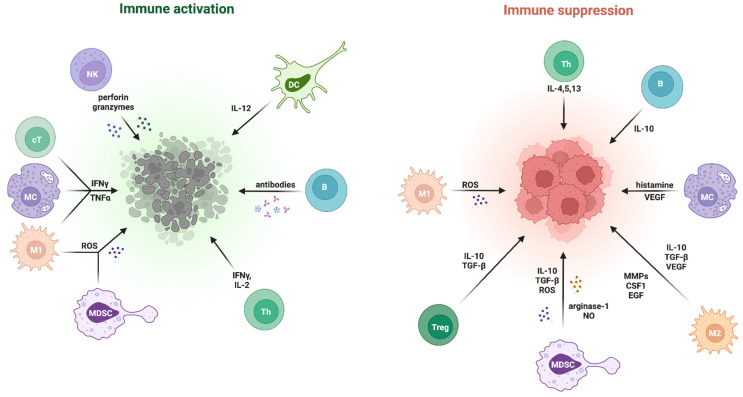
Tumour microenvironment cells and their immunostimulatory and immunosuppressive factors.

TME in breast cancer functions as a balance system that determines how well the body fights cancer based on the strength of brakes and accelerators that use receptor–ligand interactions, soluble factors, and cell-based programmes. The second PD-1 ligand, known as PD-L2, operates as a braking mechanism in cancer treatment because it exists primarily on antigen-presenting cells and shows enhanced binding to PD-1 compared to PD-L1, while its expression in breast cancer cells and immune cells follows different patterns than PD-L1 [87,88,89]. The immune cells within tumours express multiple checkpoint molecules, including LAG-3, which binds to major histocompatibility complex (MHC) class II and fibrinogen-like protein 1, and T cell immunoreceptor with Ig and TIGIT, which competes with cluster of differentiation (CD) 226 for CD155/CD112 binding sites and activates ITIM/ITT-like signalling motifs and T cell immunoglobulin and mucin-domain containing-3, which responds to galectin-9 and also interacts with carcinoembryonic antigen-related cell adhesion molecule 1. These checkpoint molecules reduce TIL function by suppressing cell growth, cytokine production, and cell-killing ability even when PD-1 blocking occurs. CTLA-4 functions as a marker for Treg and TILs, which blocks early T cell activation through two mechanisms: it competes with CD28 for CD80/CD86 binding sites, and it removes these ligands from APCs through transendocytosis [90,91]. The TME’s acidic environment enables the V-domain Ig suppressor of T cell activation to bind to P-selectin glycoprotein ligand-1 (PSGL-1) and V-set and immunoglobulin domain-containing protein 3 (VSIG-3) on myeloid and endothelial cells [92,93], while the CD47-SIRPα pathway prevents phagocytosis and cross-presentation. The immune checkpoint Natural Killer Group 2, member A (NKG2A) on natural killer cells-NK/CD8^+^ cells is controlled by human leukocyte antigen (HLA)-E [94], and B7-H3/B7-H4 expression occurs throughout the body to create ‘cold/armoured-cold’ states [95,96]. The TGF-β signal promotes lymphocyte movement from tumour nests while indoleamine 2,3-dioxygenase 1 (IDO1) suppresses effectors through kynurenine pathway activation, and CD39/CD73 produces adenosine, which activates A2A/A2B receptors to reduce cytotoxic activity [97,98,99]. Reactive oxygen and nitrogen species (ROS) act in two ways in the TME: classically activated M1 macrophages generate tumouricidal ROS that support antitumour immunity [100], whereas MDSCs produce high peroxynitrite that nitrates chemokines and TCR-associated proteins, limiting CD8^+^ T cell infiltration and signalling [101,102]. The activation of CD8^+^ T cells by BATF3-dependent cDC1s through type I IFN priming leads to strong cross-presentation and CD8^+^ T cell licensing. The downstream activation of CD28, ICOS, and OX40 and 4-1BB costimulatory molecules maintains both the survival and functional capabilities of T cells [103,104,105]. The IFN-γ-CXCR3 axis, mediated through chemokines CXCL9/CXCL10, guides effectors toward tumours while promoting an inflamed tumour environment [106]. The process of immunogenic cancer-cell death enhances dendritic cell maturation and T cell diversity through calreticulin exposure and adenosine triphosphate (ATP) and high-mobility group box 1 (HMGB1) release and MHC-I expression enhancement [107,108,109]. The immune response maintains antigen presentation and recruitment through IFN-γ-driven programmes, but these programmes also lead to adaptive resistance by promoting PD-L1 expression, thus creating a dual activating and inhibitory signal environment in individual tumours [110]. The final visible TME pattern of the immune desert or immune-excluded or inflamed status emerges from the balance between opposing forces that operate within local and regional and metastatic breast cancer areas [48]. The relationships between effector and suppressive immune cells in the tumor microenvironment (TME) are shown in Figure 4.

## 3. Immune Checkpoint Inhibitors and Their Role in Breast Cancer Treatment

### 3.1. Cancer Resistance to Immune Response

The ability of cancer cells to evade immune surveillance represents a fundamental element of tumour progression and a major barrier to effective immunotherapy. The concept of cancer immunoediting, introduced by Schreiber and later refined, describes the dynamic interaction between the immune system and malignant cells through three stages: elimination, equilibrium, and escape [111]. Within this framework, resistance to immune control can be classified as primary or secondary, each characterised by distinct mechanisms and clinical implications.

Primary resistance refers to tumours that are insensitive to immune control from the outset, meaning that immune-evasive mechanisms are present before the immune system or immunotherapeutic intervention can eliminate a substantial proportion of malignant cells. In the context of immunoediting, primary resistance typically corresponds to failure during the elimination phase and is associated with reduced tumour immunogenicity or defects in antigen presentation that prevent immune recognition of transformed cells. Low immunogenicity—resulting from the absence or low expression of strong neoantigens—limits effector T cell recruitment and activation, while defects in MHC class I/II pathways or antigen-processing machinery impair proper antigen presentation to T cells [112]. In addition, immunosuppressive cell populations, such as MDSCs and Tregs, together with unfavourable metabolic conditions, including hypoxia and acidosis, in the tumour microenvironment, hinder initial immune activation [113]. For tumours with primary resistance, therapeutic strategies should therefore focus on enhancing tumour immunogenicity, restoring or strengthening antigen presentation, and modulating the tumour microenvironment. Selecting such interventions requires pre-treatment molecular diagnostics tailored to identify specific mechanisms of resistance.

Secondary resistance, in contrast, develops after an initial immune response or therapeutic intervention, when a tumour that was initially controlled or regressing acquires mechanisms that enable renewed growth despite an active immune system. In the immunoediting model, this corresponds to the escape phase and is often preceded by a prolonged equilibrium period during which clonal selection favours cells capable of surviving under immune pressure [112,114]. Acquired resistance is driven by the emergence of clones harbouring mutations that allow immune evasion—such as antigen loss or alterations in antigen-presentation pathways—as well as amplification of immunosuppressive mechanisms, including the expression of inhibitory ligands such as PD-L1 and the secretion of suppressive factors like TGF-β, which impair effector T cell function [114,115]. Furthermore, senescent cells within the tumour microenvironment can release a senescence-associated secretory phenotype (SASP) with both pro-inflammatory and immunosuppressive activity, further promoting immune evasion and tumour progression [116].

Clinically, secondary resistance manifests as relapse or progression after an initial response to immunotherapy, necessitating long-term molecular and immunological monitoring, including tracking of PD-L1 expression dynamics, neoantigen profiles, and clonal sequencing. Computational modelling is increasingly used to quantitatively assess the degree of immune resistance and predict treatment outcomes [117]. Therapeutic strategies targeting secondary resistance include combination treatment regimens, restoration of antigen presentation, and novel counter-immunoediting approaches aimed at reversing immune escape and re-establishing immune control [115]. Determining whether resistance is primary or secondary is critical for selecting appropriate therapeutic strategies and designing clinical trials, and integrating molecular, immunological, and computational data is essential for precise identification of resistance mechanisms and the development of effective, personalised interventions [111,116,117].

### 3.2. Detailed Mechanism of Drug Action

**Figure 5:**
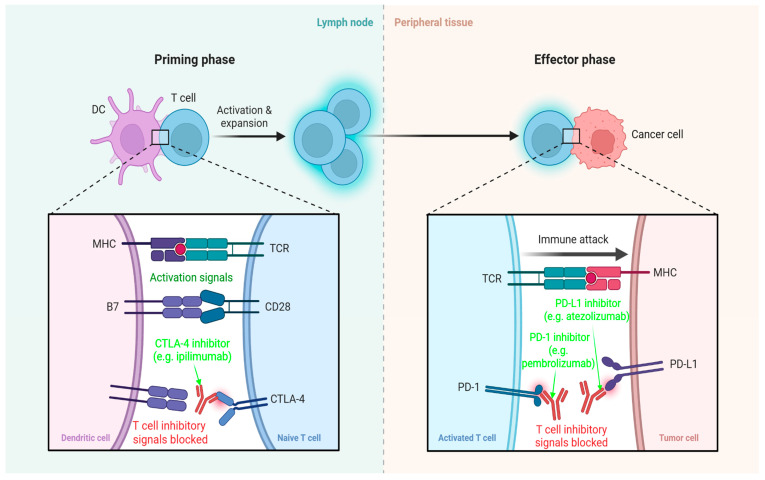
Mechanism of Action-PD-1, PDL-1 and CTLA-4 inhibitors. Created with BioRender.com.

ICIs are monoclonal antibodies that restore antitumour immunity by antagonising physiological inhibitory signals that limit T cell activation. The principal checkpoints exploited therapeutically in breast cancer are programmed cell death protein 1 (PD-1), its ligand PD-L1, and CTLA-4; blockade of these receptors reactivates exhausted effector T cells and enhances cytotoxic functions [118]. Under physiological conditions, the interaction of PD-1 expressed on activated T lymphocytes binds PD-L1/PD-L2 on antigen-presenting cells (APCs) or tumour cells, leading to the recruitment of the phosphatase SHP2, which dephosphorylates proximal T cell receptor (TCR). This process suppresses proximal TCR signalling intermediates, thereby reducing proliferation, cytokine production, and cytotoxicity. Similarly, CTLA-4, expressed on T cells during early stages of activation, competes with the costimulatory receptor CD28 for binding to CD80/CD86 on APCs. Engagement of CTLA-4 transmits inhibitory signals that dampen T cell priming in secondary lymphoid tissues, thus contributing to peripheral tolerance [119,120]. Tumour cells frequently exploit these regulatory mechanisms by upregulating PD-L1, often in response to inflammatory cytokines such as interferon-γ. This creates an immunosuppressive microenvironment that promotes T cell exhaustion and facilitates tumour progression despite the presence of immune infiltrates [118]. Antibodies targeting PD-1 (e.g., pembrolizumab, nivolumab) or PD-L1 (e.g., atezolizumab, durvalumab, avelumab) restore TCR signalling, reactivating cytotoxic lymphocytes, enhancing their proliferation, and restoring the secretion of effector cytokines such as interferon-γ and tumour necrosis factor-α [121]. In TNBC, a relatively inflamed tumour microenvironment marked by higher tumour-infiltrating lymphocyte (TIL) density and a higher neoantigen load renders checkpoint blockade particularly effective in selected patients. Large, randomised trials, most notably KEYNOTE-522 and KEYNOTE-355, have demonstrated the clinical benefit of pembrolizumab in distinct TNBC settings. The KEYNOTE-522 trial evaluated pembrolizumab with neoadjuvant chemotherapy in early-stage TNBC, showing a significant improvement in event-free survival (EFS), regardless of PD-L1 status. In contrast, the KEYNOTE-355 study, conducted in patients with metastatic or unresectable locally advanced TNBC, demonstrated that pembrolizumab combined with chemotherapy significantly improved progression-free survival (PFS) and overall survival (OS) in PD-L1-positive tumours, specifically those with a combined positive score (CPS) ≥ 10. On this basis, pembrolizumab has become the most widely adopted ICI in TNBC, establishing its role as a standard of care in both early and advanced disease settings [122,123]. Anti-CTLA-4 agents (e.g., ipilimumab) enhance T cell priming by blocking negative costimulatory signals in secondary lymphoid tissues. However, both clinical experience and preclinical studies in breast cancer demonstrate that CTLA-4 blockade as a monotherapy yields only limited antitumour activity. Consequently, its therapeutic benefit has been observed primarily in combination regimens, where synergistic effects with other immunotherapeutic or targeted strategies can be achieved [118,120]. These mechanisms have been depicted in Figure 5 below.

### 3.3. Why ICIs Are Only Used in Triple-Negative Breast Cancer

The preferential use of ICIs in TNBC is explained by fundamental differences in tumour biology and immune microenvironment across breast cancer subtypes. TNBC exhibits a relatively ‘inflamed’ phenotype characterised by higher tumour mutational burden (TMB) and neoantigen load, increased density of TILs, and more frequent PD-L1 expression in tumour and immune cells; these features create a permissive context for reinvigoration of antitumour T cell responses following PD-1/PD-L1 blockade [124]. The previously mentioned KEYNOTE clinical trials clearly demonstrate that the addition of pembrolizumab to chemotherapy in PD-L1-positive disease improves both event-free and OS, providing clinical validation [123]. In contrast, hormone-receptor-positive (HR+) and HER2-positive breast cancers generally exhibit a ‘cold’ immune microenvironment, characterised by lower TMB, sparse TIL infiltration, and less consistent PD-L1 expression [125,126]. These features limit the effectiveness of immune checkpoint blockade as monotherapy, as the preexisting antitumour immune response is often insufficient to be reinvigorated solely by PD-1/PD-L1 inhibition. Moreover, immune evasion in these subtypes is frequently mediated by intrinsic oncogenic pathways and alternative immunosuppressive mechanisms, which are less dependent on PD-1/PD-L1 signalling [126,127]. Consequently, ICIs have shown minimal single-agent activity in HR+ and HER2+ tumours, and their use in these subtypes remains largely investigational [126,128]. The success of ICIs in TNBC has driven efforts to enhance immunogenicity in resistant breast cancer subtypes through combinations with chemotherapy, targeted or anti-angiogenic agents, and emerging checkpoint inhibitors (e.g., LAG-3, TIGIT) [127,129]. Early studies suggest that these strategies may enhance antigen release, stimulate dendritic cell activation, and reduce immunosuppression, potentially broadening response rates beyond PD-L1-positive TNBC [127]. While ICIs show clear benefit in TNBC due to its inflamed microenvironment, their application in HR^+^ and HER2^+^ tumours will likely depend on rational combinations or novel immunomodulatory approaches.

### 3.4. Application to Specific Types of Breast Cancer

The application of ICIs for breast cancer treatment depends on the specific type of cancer. TNBC patients receive pembrolizumab as part of their peri-operative care, following neoadjuvant chemotherapy, and undergo post-operative treatment, without requiring PD-L1 testing, to achieve improved survival outcomes [123,130]. The treatment of metastatic cancer with ICIs requires patients to have positive PD-L1 results, as determined by the specific assay used for testing. The pembrolizumab treatment in KEYNOTE-355 requires patients to have a combined positive score (CPS) of 10 or higher [43], while atezolizumab in IMpassion130 requires an IC score of 1% or higher [131]. The CPS and IC tests identify different patient groups and should not be used as equivalent measures [132]. The experimental nature of immunotherapy for HR+/HER2− disease exists, but scientists are evaluating its potential as a tumour-agnostic therapy for patients who have MSI-H/dMMR or TMB-H at least 10 mut/Mb [130,133]. The treatment of HER2-positive breast cancer lacks any proven indications because researchers have not achieved long-lasting benefits when they tried to use ICIs together with anti-HER2 therapy, as shown in the KATE2 study [134]. The biological rationale for ICI treatment exists in metaplastic carcinoma and other ‘inflamed’ phenotypes, as these tumours show high PD-L1 expression and elevated TILs although most treatment occurs within clinical trials [135].

### 3.5. Dual Checkpoint Inhibition: Early Signals and Clinical Context

Research on dual immune checkpoint blockade in breast cancer has identified three primary therapeutic approaches. The combination of PD-L1 and CTLA-4 showed promising results in a single-arm pilot study of durvalumab and tremelimumab for HER2-negative metastatic breast cancer patients, achieving a 17% overall response rate. Notably, this response rate was higher in TNBC patients, but no responses were observed in ER-positive patients [136]. The dual ICI treatment in a small ‘window-of-opportunity’ study for HR+/HER2 patients resulted in limited clinical activity with a pCR rate of 12.5%, and the study ended prematurely due to adverse effects and suspected disease progression [137]. The PD-1 + LAG-3 combination demonstrated feasibility and early signs of immunological response in a short neoadjuvant TNBC treatment for patients with high TILs, but mature EFS/OS results remain unavailable [138]. The PD-1 + CTLA-4 combination represents the most studied axis in this context. The BELLINI trial administered nivolumab with low-dose ipilimumab to patients with TIL at least 50% for six weeks, which resulted in MPR 53% and pCR 33% but caused at least three adverse events in 17% of patients and endocrine toxicities that lasted for extended periods in 57% of participants [139]. The combination of nivolumab 240 mg every 2 weeks with ipilimumab 1 mg/kg every 6 weeks in DART/SWOG S1609 for metaplastic mBC resulted in ORR 18% with three durable responses lasting 28–34+ months, while PFS reached 2 months, and OS reached 12 months, and all three responders developed adrenal insufficiency [140].

### 3.6. Therapy Effectiveness

Studies on the effectiveness of breast cancer therapy using ICIs focus primarily on the use of PD-1 inhibitors in cases of TNBC. Most of the research concerns advanced, metastatic-stage disease [118]. The KEYNOTE-012 study from 2016, a phase Ib trial, involved administering pembrolizumab (a PD-1 inhibitor) at a dose of 10 mg/kg body weight every two weeks to patients with advanced, PD-L1-positive TNBC. The study showed that among the 27 evaluable patients, the overall response rate was 18.5%. The conclusions drawn from this trial indicated that pembrolizumab demonstrated clinical activity and a potentially acceptable safety profile in advanced TNBC [141]. The phase III KEYNOTE-119 study, conducted between 2015 and 2017 in patients with advanced TNBC, demonstrated no significant improvement in OS among those treated with pembrolizumab compared to those receiving standard chemotherapy—the median OS was 9.9 months and 10.8 months, respectively [142]. Studies have also been conducted on combination therapy using atezolizumab in conjunction with nab-paclitaxel and paclitaxel in patients with advanced TNBC. Although no statistically significant improvement in OS was observed in the entire study population, patients with ≥1% of cells expressing PD-L1 showed an increase in median OS [143,144]. The first evidence of a significant improvement in PFS was observed in the phase III KEYNOTE-355 study, conducted between 2017 and 2018. This study demonstrated that patients with metastatic TNBC treated with pembrolizumab in combination with chemotherapy had better outcomes compared to those receiving chemotherapy with placebo [122]. As a result, this therapy was approved by the U.S. Food and Drug Administration (FDA) for the treatment of advanced PD-L1-positive TNBC [118]. Another study demonstrating the efficacy of ICIs in advanced breast cancer was the MEDIOLA trial, in which patients with metastatic *BRCA*-mutated breast cancer received a combination of olaparib (a poly(ADP-ribose) polymerase inhibitor) and durvalumab (a PD-L1 inhibitor) [145]. The results showed a high disease control rate of 80% at 12 weeks and 50% at 28 weeks. In ER+ and HER2+ breast cancers, studies have shown a poor response to treatment or increased hepatotoxicity and pneumotoxicity [118]. Overall, in advanced metastatic TNBC, the use of PD-L1 inhibitors is an effective therapy; however, it typically requires combination with conventional chemotherapy.

In early-stage TNBC breast cancers, studies have shown a relatively good response to treatment with PD-L1 inhibitors. The KEYNOTE-522 study, which included patients treated with pembrolizumab, demonstrated a higher pathological complete response (pCR) rate with neoadjuvant therapy, as well as longer event-free survival (EFS) with adjuvant therapy [146,147]. Other studies, such as IMpassion031, have investigated the efficacy of combination therapy involving nab-paclitaxel and atezolizumab with chemotherapy. The results demonstrated an increased pathological complete response (pCR) rate, rising from 41% to 58% [148]. Although the combination of nab-paclitaxel and durvalumab in neoadjuvant therapy did not result in a higher pCR rate, it was associated with improved 3-year OS) [149]. In early-stage luminal breast cancer, the addition of PD-1 inhibitors may increase the pCR; however, this effect is observed primarily in the luminal B subtype and not consistently across all cases [81,150]. The latest clinical trials, KEYNOTE-756 and CheckMate 7FL, have shown that adding nivolumab or pembrolizumab to neoadjuvant therapy in early ER+/HER2− breast cancer improves the pCR rate [151]. In conclusion, similar to advanced breast cancer, the greatest clinical benefit from PD-1/PD-L1 pathway inhibition in early-stage disease is achieved in patients with triple-negative breast cancer (TNBC). However, recent study results reveal some effectiveness in selected luminal breast cancer subtypes, suggesting the potential for future application of these therapies in this situation.

## 4. Clinical Trials Landscape and Regulatory Approvals

### 4.1. Landmark Trials

Pembrolizumab is currently the sole immunotherapy agent with FDA approval for the treatment of breast cancer. This approval was based on the results of two landmark studies: KEYNOTE-522 and KEYNOTE-355.

The KEYNOTE 522 (NCT03036488) publication became the basis for FDA approval of pembrolizumab in neoadjuvant therapy in combination with chemotherapy, followed by adjuvant therapy for early TNBC. It was a phase III, double-blind, randomised (2:1) study in patients with previously untreated, early-stage TNBC, stage II–III. KEYNOTE 522 demonstrated multiple benefits of the investigated treatment regimen: higher pCR [146], EFS [147], and OS [123].

The authors of the KEYNOTE 355 (NCT02819518) study aimed at estimating the PFS and OS of patients on pembrolizumab in conjunction with chemotherapy. The study was performed on patients with advanced triple-negative breast cancer whose tumours expressed programmed death-ligand 1 (PD-L1) with a combined positive score of 10 or more. It was a stage III trial, double-blind, randomised (2:1) study. The addition of pembrolizumab to chemotherapy resulted in significantly longer OS rate, and in patients than chemotherapy alone [43].

### 4.2. FDA/EMA Approvals and Criteria

Atezolizumab was approved by the FDA in 2019 based on the IMpassion130 trial (NCT02425891). This was the first-ever approval of immunotherapy in the treatment of breast cancer. However, following the negative results of the IMpassion131 trial (NCT03125902), the manufacturer voluntarily withdrew it in agreement with the FDA in the United States. The latter study evaluated the efficacy and safety of combining atezolizumab with paclitaxel in patients with previously untreated, locally advanced, or metastatic TNBC. The results showed that such a combination did not improve either PFS or OS compared to paclitaxel alone. Table 2 and Table 3 show drugs already approved and in ongoing clinical trials, in breast cancer approval, including their availability in selected countries.

### 4.3. Ongoing Clinical Trials and Trial Designs

Although the combination of atezolizumab and paclitaxel was proven ineffective in IMpassion131, recent studies present promising results for a different combination of agents. In the phase II TBCRC 043 trial (NCT03206203), the addition of atezolizumab to carboplatin improved PFS and overall survival (OS) in patients with metastatic TNBC [152]. Tropion-Breast03 is one of the recent studies investigating the efficacy and safety of TNBC therapy based on the combination of Dato-DXd with durvalumab. ADCs, similarly to the previously described cytostatics, are capable of inducing ICD, thereby enhancing the efficacy of checkpoint inhibitors [162]. However, it seems that in the future, ADCs may be more suitable for this purpose because, due to their structure, they limit systemic toxicity and ensure drug activity within the tumour microenvironment [163]. The drug combination discussed in the study is likely also beneficial due to increased infiltration of cytotoxic T lymphocytes [164].

A promising future treatment strategy also appears to be dual checkpoint blockade through the combination of a CTLA-4 inhibitor with a PD-1/PD-L1 inhibitor. The NIMBUS study (NCT03789110) tested the combination of nivolumab with ipilimumab in hypermutated HER2-negative metastatic breast cancer [157]. Such a combination induces a synergistic immune response in T cell activation and enhances antitumour immunity, particularly in tumours with a high mutational burden [165].

Pembrolizumab is not the only PD-1 inhibitor that, when used in conjunction with chemotherapy, shows promise in treating TNBC. Agents such as camrelizumab and toripalimab demonstrate promising results in phase III trials, achieving positive outcomes in PFS and OS of patients [155,166].

## 5. Predictive and Prognostic Biomarkers for Checkpoint Inhibitor Response

### 5.1. PD-L1 Expression: Methods of Detection, Thresholds, and Heterogeneity

PD-L1 expression is assessed on formalin-fixed, paraffin-embedded tumour tissue sections using immunohistochemical (IHC) assays [167]. The most commonly used assays include SP142, SP263, 22C3, and 28-8 [168]. Studies have shown significant differences among these assays, with 22C3 and SP263 exhibiting higher sensitivity compared to SP142 [169]. Another challenge lies in the interpretation of test results; however, appropriate training of pathologists has been shown to substantially reduce inter-observer variability [170]. PD-L1 expression is evaluated separately in immune cells (IC) and tumour cells (TC). IC assessment considers the percentage of tumour area covered by stained inflammatory infiltrating cells, regardless of staining intensity. TC assessment evaluates the percentage of viable tumour cells stained, with both partial and complete staining considered positive [171]. A third indicator, which incorporates both IC and TC, is the CPS. CPS is calculated by adding the number of stained IC and stained TC, dividing this sum by the total number of viable tumour cells, and multiplying by 100. CPS reflects the overall level of PD-L1 expression within the tumour microenvironment. The test is considered positive if the CPS is 1 or higher, and negative if it is less than 1 [172]. PD-L1 expression in breast cancer demonstrates both spatial and temporal heterogeneity. It has been reported that PD-L1 expression may differ between primary tumours and metastatic sites, indicating that patients with PD-L1-negative primary tumours may still qualify for immunotherapy depending on PD-L1 expression in metastatic lesions [173]. It should also be noted that tumours treated with neoadjuvant therapy tend to show lower PD-L1 expression compared to pretreatment levels, which may contribute to loss of response to immunotherapy and further tumour progression [174]. ICIs targeting PD-L1 have demonstrated therapeutic efficacy in the management of TNBC. Studies indicate that the expression of PD-L1 in tumour cells or within the immune infiltrate is associated with a better response to checkpoint inhibitor therapy. Therefore, a positive IHC test result may suggest that the patient is more likely to respond to treatment, which in turn correlates with a more favourable prognosis [167].

### 5.2. Tumour Mutational Burden

Tumour Mutational Burden (TMB) is defined as the number of somatic mutations per megabase of the sequenced genome [175]. Compared with other malignancies, breast cancer is characterised by a moderate TMB, with a 2020 study involving 3.969 patients reporting a median value of 2.62 mut/Mb for this cancer type. It is important to note that TMB varies significantly among breast cancer subtypes and that metastatic tumours often display higher TMB than primary tumours [176]. TNBC is generally associated with a higher mutation load compared with other breast cancer subtypes [177]. Although certain studies link higher TMB to poorer outcomes in breast cancer, others report no significant correlation. This lack of consistency limits the reliability of TMB as a prognostic marker for this cancer type. A recent meta-analysis showed that TMB is not suitable for use as a prognostic marker in breast cancer. However, high TMB may be associated with longer survival in patients treated with immunotherapy [178]. High TMB is hypothesised to be linked to improved responsiveness to immunotherapy, thereby serving as a potentially favourable predictive marker [176]. The relationship between PD-L1 expression and TMB remains unclear. Available evidence suggests that PD-L1 and TMB may function as independent predictive markers for response to immune checkpoint blockade [177]. A 2020 meta-analysis demonstrated that high TMB in breast cancer may be associated with longer survival in patients treated with checkpoint inhibitors, but this correlation was not observed in patients receiving other forms of therapy [178]. Beyond breast cancer, high TMB has also been shown to correlate with improved response to checkpoint inhibitors in other malignancies, such as melanoma and lung cancer. Higher TMB can lead to the production of more neoantigens that the immune system recognises as foreign, making the tumour more visible to the immune system, and more susceptible to the effects of immunotherapy [179]. One of the major challenges in introducing TMB into routine clinical practice is the lack of standardised measurement methods [177]. In conclusion, while TMB is unsuitable as a prognostic marker in breast cancer, it holds value as a predictive marker for response to checkpoint inhibitor therapy.

### 5.3. Microsatellite Instability

Microsatellites are short, repetitive DNA sequences that, while usually stable, are prone to replication errors. In normal cells, these mistakes are corrected by the mismatch repair (MMR) system. When MMR is defective (dMMR), replication errors persist, causing frameshift mutations and leading to microsatellite instability (MSI) [180,181]. In 2017, the FDA granted accelerated approval of pembrolizumab for adults and children with unresectable or metastatic MSI-H or dMMR solid tumours that have progressed after prior therapy and lack other treatment options, including MSI-H/dMMR colorectal cancer resistant to fluoropyrimidine, oxaliplatin, and irinotecan [182]. Then, on 26 July 2021, the FDA approved pembrolizumab with chemotherapy for high-risk, early-stage triple-negative breast cancer (TNBC) as neoadjuvant therapy, followed by single-agent adjuvant use after surgery. The FDA also gave full approval for pembrolizumab plus chemotherapy in locally recurrent, unresectable, or metastatic TNBC with PD-L1 expression (CPS ≥ 10), converting the accelerated approval first granted in November 2020 [183]. While MSI-H/dMMR breast cancers are rare (less than 2% of TNBC cases are MSI-H/dMMR), this approval technically covers them if identified. As of August 2025, dostarlimab—an anti-PD-1 monoclonal antibody—has been used in breast cancer treatment to a certain extent, although it does not have a breast-cancer-specific FDA approval. Phase 2 TBCRC-056 trial (NCT04584255) investigated niraparib + dostarlimab-gxly treatment as a therapy in *BRCA*/*PALB2* mutations and ER+/HER2− breast cancer [184].

### 5.4. Gene Expression Signatures

A gene expression signature refers to a defined set of genes whose coordinated expression pattern captures a particular biological condition, cellular state, or response to a stimulus. Such signatures are widely used to characterise disease phenotypes, stratify tumour subtypes, and predict therapeutic outcomes [185]. Interferon-gamma (IFN-γ) is a cytokine primarily associated with the oncological immune response. It is secreted by activated T cells and NK cells and plays a key role in coordinating immune responses but can also trigger negative feedback that weakens antitumour activity. It achieves this by upregulating PD-L1, PD-L2, and IDO1, which suppress T cell function in the tumour microenvironment [110]. An IFN-γ–related gene expression signature is a set of genes whose expression levels change in response to interferon-gamma (IFN-γ). IFN-γ activates the JAK–STAT pathway, leading to transcription of interferon-stimulated genes—a curated subset of these genes can be measured to provide a ‘signature’ that correlates with immune activation, inflammation or responsiveness to immunotherapy in cancer [186,187]. It can predict patient prognosis, inform the effectiveness of immunotherapies like PD-1 checkpoint blockade, and indicate the immune characteristics of a tumour’s microenvironment [110,186]. In the KEYNOTE-001 trial, biopsies from 19 patients with metastatic melanoma were analyzed to explore immune gene expression associated with pembrolizumab response. A 10-gene IFN-γ signature (*IFNG*, *STAT1*, *CCR5*, *CXCL9*, *CXCL10*, *CXCL11*, *IDO1*, *PRF1*, *GZMA*, and *MHCII HLA-DRA*) was identified that distinguished responders from non-responders. In a second cohort of the same study, this set has been expanded into a 28-gene set. Both signatures are significantly associated with response and PFS, highlighting IFN-γ–driven immune activity as key to pembrolizumab’s efficiency in cancer [110]. The KEYNOTE-173 trial evaluated tumour microenvironment associated with the response to the combination of pembrolizumab and chemotherapy administered as a neoadjuvant treatment in TNBC patients. The study assessed the T cell-inflamed gene expression profile (Tcell_inf GEP) and 10 other gene expression profiles. Although Tcell_inf GEP suggested enhanced immunologic activity among responding patients, it was not a strong predictor of pCR in this study—AUROC 0.55 (95% CI 0.25–0.85). On the other hand, dendritic cell and macrophage signatures were associated with a higher likelihood of pCR [188]. In KEYNOTE-522, the impact of combining chemotherapy with pembrolizumab immunotherapy was also evaluated in patients with early-stage TNBC in the neoadjuvant setting. The findings of this study suggest that treatment response may be associated with Tcell_inf GEP signature and TMB. It is worth noting that Tcell_inf GEP was consistently associated with higher pCR and longer EFS in both pembrolizumab +/− chemotherapy groups, while TMB was predictive only with pembrolizumab [189]. Although clinical trials such as KEYNOTE-173 and KEYNOTE-522 have demonstrated an association between the Tcell_inf GEP signature and treatment response in TNBC patient groups, its predictive value requires further investigation.

## 6. Negative Aspects of Checkpoint Inhibitor Therapy: Adverse Effects

Checkpoint Inhibitor Therapy is associated with numerous adverse effects, many of which can be potentially severe. The long-term complications associated with this therapy remain insufficiently characterised, primarily due to the relatively recent introduction of checkpoint inhibitors into clinical practice. Consequently, comprehensive studies addressing late adverse effects are still lacking [190]. A systematic review by Jayathilaka et al., encompassing 293 clinical trials conducted between 2017 and 2021, demonstrated that up to 40% of patients undergoing this therapy experience some form of immune-related adverse event, with serious adverse events occurring in 19.7% of cases. This incidence rises to 45.7% in the context of combination therapy involving two types of checkpoint inhibitors [191]. Cutaneous immune-related adverse events (irAEs) are the most common and typically the earliest side effects of ICIs. Particular attention should be given to maculopapular rash, pruritus, and various dermatoses, as these represent the most common manifestations within the category of skin disorders [192]. The gastrointestinal system, including the liver and pancreas, also constitutes a frequent site of complications. Common and often persistent symptoms include nausea, vomiting, diarrhea, and abdominal pain. In some cases, patients may present with severe, potentially life-threatening manifestations such as colitis, hepatitis, pancreatitis, or bowel perforation [193]. ICI therapy has also been associated with endocrine disorders, including both hyperthyroidism, and hypothyroidism, diabetes mellitus, and hypophysis [194,195]. Additional reported adverse effects involve the cardiovascular system, respiratory system, renal, and neurological disorders [194]. Different ICI therapies are associated with varying profiles of organ-specific adverse effects. For instance, patients treated with PD-1/PD-L1 inhibitors most commonly develop cutaneous conditions, immune-mediated pneumonitis, and musculoskeletal pain. In contrast, CTLA-4 inhibitors are more frequently associated with colitis, maculopapular rash, and hypophysitis [192]. Complete prevention of adverse events is not yet possible; however, several strategies can reduce risk and enable early detection of complications. A study by Wang et al. identified female sex, antibiotic use, elevated neutrophil to lymphocyte ratio, and higher baseline circulating TC levels as predictors of irAE occurrence [196]. Genetic testing may be useful, as certain HLA-DR alleles increase the risk of specific irAEs, such as diabetes and reactive arthritis [190,197]. Studies on gut microbiota composition and fecal microbiota transplantation also show promise. Milder adverse events often require only symptomatic treatment or temporary discontinuation of immunotherapy. Severe irAEs are typically managed with glucocorticoids, while targeted biologic agents are used in steroid-refractory cases. Detailed management algorithms have been developed and published by the European Society for Medical Oncology [197,198].

## 7. Contraindications

Although ICI therapy may offer the potential to improve both the duration and quality of life in patients with various types of cancer, there are numerous conditions and circumstances in which the use of this class of drugs is contraindicated. High doses of corticosteroids are generally contraindicated during ICI therapy due to their potential to diminish antitumoural effectiveness in advanced melanoma patients treated with anti-PD-1 monotherapy [199]. Patients with coexisting preexisting autoimmune diseases have been excluded from cancer immunotherapy clinical trials due to concerns regarding potential flare-up of their underlying autoimmune conditions and the consequent development of severe treatment-related adverse events [200]. A meta-analysis by W. Xie et al. demonstrated that active autoimmune diseases may not constitute an absolute contraindication to the use of ICIs. Nonetheless, their use is associated with an increased incidence of adverse events in patients with rheumatoid arthritis. It is worth noting that although these adverse effects were generally mild, a thorough evaluation of potential benefits and risks should be conducted on an individual basis for each patient [201]. Similarly, severe viral infections such as HIV and HBV/HCV, pregnancy, and organ transplantation were exclusion criteria in the clinical trials of ICIs. As a result, comprehensive data on their safety in the aforementioned patient populations remain unavailable [202,203,204].

## 8. Future Perspectives

Despite continuous advances in oncology and immunotherapy over recent years, breast cancer still represents a major therapeutic challenge, with many questions remaining unanswered. Further progress requires coordinated efforts across the entire scientific community. Advances in cancer treatment largely depend on deepening our understanding of the biological mechanisms of the disease, as well as on the development of new diagnostic and therapeutic tools. In the future, the identification of novel prognostic markers and the development of sensitive and standardised IHC assays may play a particularly important role, enabling a more precise assessment of prognosis and treatment efficacy. Artificial intelligence will also play an increasingly important role in diagnosis and treatment. Its application in the evaluation of histological specimens may significantly improve the accuracy of IHC-based diagnostics and enable partial automation of the diagnostic process. At the same time, it is essential to further expand knowledge regarding both gene expression in cancer cells and signalling pathways, as well as mechanisms by which cancer cells evade immune surveillance. This knowledge forms the foundation for developing new therapeutic targets for immunotherapy and for more informed design of preclinical and clinical studies. It is also crucial to determine the effectiveness of immunotherapy in different breast cancer subtypes, as well as to conduct studies on combining immunotherapy with other treatment options. Confirmation of the effectiveness of combining hormone therapy with PD-1 inhibitors in ER+ metastatic breast cancer is also necessary. The development of therapies must additionally include the refinement of minimally invasive surgical techniques. Furthermore, emphasis should be placed on the necessity of personalised treatment, with a particular focus on tailoring therapies to each patient to achieve the best possible outcomes with minimal risk. With the increasing number of immunotherapy regimens being applied, greater attention must be paid to treatment safety. In the future, it will be essential to conduct studies on the late adverse effects of immunotherapy, which are currently lacking due to the relatively recent introduction of this therapeutic approach and the limited data available in this area. Assessing the long-term safety profile is crucial for determining the true clinical value of new therapies. In conclusion, future research should focus on the parallel development of diagnostic tools, the deepening of biological knowledge, the refinement of therapies, and the evaluation of their safety.

## 9. Conclusions

The introduction of ICIs has transformed treatment strategies for TNBC, a highly aggressive and treatment-resistant subtype. Combining ICIs with chemotherapy has resulted in improved response rates and long-term outcomes in specific patient groups. Notably, agents targeting the PD-1/PD-L1 pathways, including pembrolizumab and atezolizumab, have demonstrated efficacy in both metastatic and early-stage TNBC. However, the extent of benefit is largely determined by biomarker-defined subgroups [43,146].

The TME is a critical determinant of therapeutic outcomes. Factors such as the presence of TILs, the density of TAMs, and PD-L1 expression levels are associated with the likelihood of response to ICIs. In addition to PD-L1, biomarkers including TMB, dMMR, and immune gene expression signatures are being investigated to improve patient selection and enable personalised therapy [110,205,206]. In early-stage TNBC, the addition of ICIs to neoadjuvant chemotherapy has led to higher pCR rates, which translate into improved EFS. The KEYNOTE-522 trial demonstrated that pembrolizumab significantly increased pCR and EFS rates when combined with standard chemotherapy, regardless of PD-L1 status, highlighting the potential of ICIs as part of curative treatment strategies in TNBC [146]. These results support integrating immunotherapy into earlier lines of therapy to prevent metastatic spread and improve survival.

In metastatic TNBC, the benefit of ICIs is limited. The IMpassion130 trial demonstrated a survival advantage with atezolizumab only in PD-L1-positive patients, whereas subsequent studies such as IMpassion131 did not confirm these results. These findings highlight the heterogeneity of TNBC and the complexity of immune–tumour interactions [131,207]. Consequently, PD-L1 alone may be inadequate as a predictive biomarker, indicating a need for additional markers or composite scores to guide therapy.

Despite encouraging efficacy, irAEs remain a significant limitation of ICIs. Toxicities such as pneumonitis, colitis, endocrinopathies, and dermatological complications can be severe and may necessitate treatment discontinuation or immunosuppressive therapy. Careful monitoring and early intervention are critical to balance efficacy with safety [197,198]. Moreover, long-term consequences of immune modulation remain incompletely understood and warrant ongoing surveillance in clinical practice.

Future research focuses on combining ICIs with other therapeutic modalities, such as PARP inhibitors, anti-angiogenic agents, cancer vaccines, and novel checkpoint targets including LAG-3, TIGIT, and TIM-3. These strategies aim to address both primary and acquired resistance and to improve the durability of immune responses [208,209]. Technological advances in single-cell profiling and spatial transcriptomics are anticipated to enhance understanding of immune evasion in TNBC and reveal new therapeutic targets.

In summary, ICIs have become integral to the management of TNBC, especially in the neoadjuvant setting and for PD-L1-positive metastatic disease. Nevertheless, clinical benefits are variable, and there is an urgent need for predictive biomarkers beyond PD-L1. Achieving an optimal balance between efficacy and immune-related toxicity necessitates careful patient selection and management. Ongoing clinical trials and translational research are expected to broaden therapeutic options, ultimately aiming to convert TNBC into a subtype with sustained treatment responses and improved survival outcomes [131,146,197].

## Figures and Tables

**Table 1:** Summary of immunotherapy methods currently tested in breast cancer patients.

Type	Examples/Targets	Stage/Use
Checkpoint inhibitors	Pembrolizumab, Atezolizumab	Approved [43] (PD-L1 + TNBC)
Cancer vaccines	NeuVax, PVX-410	Experimental [44]
Adoptive cell therapy	HER2-CAR T, TILs	Early trials [45]
ADCs/mAbs	Trastuzumab, T-DXd, Sacituzumab govitecan	Approved [46]
Cytokines/modulators	IL-2, TLR agonists	Experimental [47]

**Table 2:** Immune checkpoint inhibitor therapies already approved and in ongoing trials (PD-1—programmed cell death protein 1, PD-L1—programmed death-ligand 1, CTLA-4—cytotoxic T-lymphocyte-associated protein 4). * Atezolizumab was approved by the FDA in 2019, then withdrawn in 2021.

Drug	Inhibition	FDA Approved	Available in EU	Stage of Clinical Trials
pembrolizumab	PD-1	Yes	Yes	already approved
atezolizumab	PD-L1	No *	Yes	II [152]
durvalumab	PD-L1	No	No	II [153], III [154]
camrelizumab	PD-1	No	No	II [155], III [156]
nivolumab	PD-1	No	No	Ib, II [140,151], II [157]
toripalimab	PD-1	No	No	II [158]
tislelizumab	PD-1	No	No	II [159]
ipilimumab	CTLA-4	No	No	Ib [160], II [140], II [157]
tremelimumab	CTLA-4	No	No	I [161], II [154]

**Table 3:** Targeted therapies including immune checkpoint inhibitors available in Poland versus selected countries, designated for breast cancer treatment. (‘+’—refunded, ‘+/−’—available but not refunded, ‘−’—not available).

Therapy Including Immune Checkpoint Inhibitors and Availability in Selected Countries	Poland	Germany	Sweden	United Kingdom	United States of America	China	Japan
Pembrolizumab	+	+	+	+	+	+	+
Atezolizumab	+/−	+	−	+/−	−	+	+
Tislelizumab	−	−	−	−	−	+	−
Camrelizumab	−	−	−	−	−	+	−

## Data Availability

No new data were created or analyzed in this study.

## Abbreviations

APCs	antigen-presenting cells
ATP	adenosine triphosphate
CD	cluster of differentiation
CPS	combined positive score
CSF1	colony stimulating factor 1
CTLA-4	cytotoxic T-lymphocyte-associated protein 4
DAMPs	damage-associated molecular patterns
dMMR	defective mismatch repair system
DNA	deoxyribonucleic acid
EFS	event-free survival
EGF	epidermal growth factor
FDA	U.S. Food and Drug Administration
FGF2	fibroblast growth factor 2
GnRH	gonadotropin-releasing hormone
HER2	human epidermal growth factor receptor 2
HIF-1	hypoxia-induced factor 1
HLA	Human leukocyte antigen
HMGB1	high-mobility group box 1
HR	hormone receptor
IC	immune cells
IDO1	Indoleamine 2,3-dioxygenase 1
IHC	immunohistochemical
IFN	interferon
irAEs	immune-related adverse events
LAG3	lymphocyte-activation gene 3
MDSCs	myeloid-derived suppressor cells
MHC	major histocompatibility complex
MMR	mismatch repair
NK	natural killer cells
NKG2A	Natural Killer Group 2, Member A
OS	overall survival
PFS	progression-free survival
pCR	pathological complete response
PD-1	programmed cell death protein 1
PD-L1	programmed death-ligand 1
PR	progesterone receptor
PSGL-1	P-selectin glycoprotein ligand-1
TAMs	tumour-associated macrophages
Tc	cytotoxic lymphocyte
TC	tumour cells
TGF-β	transforming growth factor beta
TIGIT	T cell immunoreceptor with Ig and immunoreceptor tyrosine-based inhibitory motif domains
TILs	tumour-infiltrating lymphocytes
TMB	tumour mutational burden
TME	tumour microenvironment
TNBC	triple-negative breast cancer
TNFα	tumour necrosis factor alpha
Tregs	regulatory T cells
VEGF-A	vascular endothelial growth factor A
VSIG-3	V-set and immunoglobulin domain-containing protein 3
